# The Determinants for the Enzyme Activity of Human Parvovirus B19 Phospholipase A2 (PLA2) and Its Influence on Cultured Cells

**DOI:** 10.1371/journal.pone.0061440

**Published:** 2013-04-15

**Authors:** Xuefeng Deng, Yanming Dong, Qianhui Yi, Yu Huang, Dan Zhao, Yongbo Yang, Peter Tijssen, Jianming Qiu, Kaiyu Liu, Yi Li

**Affiliations:** 1 College of Life Sciences, Central China Normal University, Wuhan, China; 2 Bioengineering Department, Wuhan Bioengineering Institute, Wuhan, China; 3 INRS-Institut Armand-Frappier, Université du Québec, Laval, Canada; 4 Department of Microbiology, Molecular Genetics and Immunology, University of Kansas Medical Center, Kansas City, Kansas, United States of America; Institut Pasteur, France

## Abstract

Human parvovirus B19 (B19V) is the causative agent of erythema infectiosum in humans. B19 infection also causes severe disease manifestations, such as chronic anemia in immunocompromised patients, aplastic crisis in patients with a high turnover rate of red blood cells, and hydrops fetalis in pregnant women. Although a secreted phospholipase A2 (PLA2) motif has been identified in the unique region of the B19V minor capsid protein VP1(VP1u), the determinants for its enzyme activity and its influences on host cells are not well understood. The purpose of this study was to investigate the contribution of the PLA2 motif and other regions of the VP1u to the PLA2 activity, to determine the cellular localization of the VP1u protein, and to examine the effects of VP1u on cellular cytokines. We found that in addition to the critical conserved and non-conserved amino acids within the VP1u PLA2 motif, amino acid residues outside the VP1u PLA2 motif are also important for the PLA2 activity. VP1u and various mutants all revealed a nucleo-cytoplasmic distribution. UT7-Epo cells treated with prokaryotic expressed VP1u or mutant proteins with PLA2 activity released a large amount of free fatty acid (FFA), and the cell morphological change occurred dramatically. However, neither free fatty acid nor cell morphology change occurred for cells treated with the mutants without PLA2 activity. The wild type and the VP1u mutants with the PLA2 activity also activated TNF-α promoter and upregulated the transcription activity of NF-κB in transfected cells. In addition, we found that the amino acids outside the PLA2 domain are critical for the viral PLA2 activity, and that these tested VP1u mutants did not affect the localization of the VP1u protein.

## Introduction

Human parvovirus B19 (B19) was first discovered in 1975 in England in the serum of a healthy blood donor [1]. It is one of two pathogenic human viruses belonging to the *Parvoviridae* family with a worldwide distribution [2]–[4]. B19V infects humans of all ages and causes several syndromes. Infection causes fifth diease in children, polyarthropathy syndrome in adults, transient aplatics crisis in patients with underlying chronic hemolytic anemia, and chronic persistent anemia in immunodeficient and immunocompromised patients [2], [4]–[7]. B19 is normally spread via the respiratory route, and blood transmission is also a common means, although the precise viral load required to initiate infection is unknown. When infection occurs during pregnancy, it may cause severe anemia and nonimmune hydrops fetalis (NIHF), which could lead to fetal damage and fetal death [4], B19V infection also has been associated with chronic and acute myocarditis [8].

The B19 genome encodes nonstructural proteins (NS1, 11 kDa and 7.5 kDa) and two capsid proteins (VP1 and VP2). The NS1 protein plays pivotal roles in the genome replication and induces apoptosis of both B19V-permissive and non-permissive cells [9]–[11]. It activates the expression of several cytokines, such as IL-6 [12]. VP1 (83 kDa) and VP2 (58 kDa) proteins are identical except for 227 amino acids (aa) at the amino-terminal end of the VP1 protein (the VP1 unique region, VP1u). A conserved PLA2-like motif (HDXXY) was identified in B19V VP1u, and several amino acids in the highly conserved domain of the VP1u share homologies to the Ca^2+^-binding loop and catalytic site of secreted PLA2. These motifs are present in the amino acid sequence of the VP1u spanning positions at amino acids from 130 to 195 [13], [14]. Mutations of critical amino acid residues in the B19 VP1u resulted in strongly reduced PLA2 activity and virus infectivity [15], [16].

Phospholipases are enzymes that hydrolyse phospholipids to generate free fatty acids and lysophospholipids [17], [18]. They are classified according to the bond cleaved in a phospholipid. Thus, PLA2 hydrolyses specifically the 2-acyl ester (*sn*-2) bond of phospholipid substrates to generate lysophospholipids and free fatty acids. However, the roles of B19 VP1u and its PLA2 motif on B19-related diseases have not been investigated [19].

Nuclear factor-κB (NF-κB) is a pivotal regulator of the immediate early pathogen response and plays an important role in promoting immune and inflammatory responses. iNOS, NO and COX-2 contributed to the pathogenesis of the inflammatory processes governed predominantly by the transcription factor NF-kB [20]. TNF-α, which contains κB binding sites in the promoter region, is also associated with various inflammatory responses. Induction of iNOS was detected in vascular endothelium cells stimulated with TNF-α. Recent studies have reported that increased expression of TNF-α, phosphorylated-p38 and iNOS were detected in ECV-304 cells treated with rabbit anti-B19 VP1u IgG [21]. In the present study, we showed that mutations not only in conserved amino acids but also in non-conserved amino acids in VP1u reduced the PLA2 activity of the VP1u. Therefore, this non-conserved region of VP1u may be important for maintaining the three dimensional structure of the VP1u to present the PLA2 activity. We found that some VP1u mutants triggered the release of fatty acids and the cell morphological change. In the current study, we also provide evidence that VP1u and VP1u mutants with the PLA2 enzymatic activity could induce the transcriptional activities of NF-κB and TNF-α.

**Table:** **Table 1.** Influences of mutations on secreted PLA2 (PLA2) activity.

Name	Mutagenesis site^a^	PLA2 activity^b^(µmol/min/ml)
VP1u(pB19-M20)	**–**	0.254
**Group I (Catalytic network)**		
VP1u(Y130A)	T to G at nt 3011	0.002
	A to C at nt 3012	
VP1u(G132A)	G to C at nt 3018	ND
VP1u(D154A)	A to C at nt 3084	ND
**Group II (Binding calcium ions)**		
VP1u(H153A)	C to G at nt 3080	0.001
	A to G at nt 3081	
VP1u(Y157F)	A to T at nt 3093	0.003
VP1u(Y168F)	A to T at nt 3126	ND
VP1u(D175A)	A to G at nt 3147	0.002
VP1u(D195A)	A to C at nt 3207	0.28
VP1u(Y157F/Y168F)	A to T at nt 3093	0.001
	A to T at nt 3126	
VP1u(Y157F/D175A)	A to T at nt 3093	0.002
	A to G at nt 3147	
**Group III (Phospholipid environment)**		
VP1u(K162R)	A to G at nt 3108	0.003
**Group IV (No-conserved)**		
VP1u(P133R)	C to G at nt 3021	0.213
VP1u(A207Y)	G to U at nt 3042	0.002
	C to A at nt 3043	
bv PLA2	**–**	0.316
PBS	**–**	0

ND means non-detected.

a Nucleotide numbers are based on the sequence of the J35 isolate (GenBank accession no. AY386330).

b Enzyme activity was measured by a colorimetric assay kit (Cayman Chemical). Protein concentrations tested: recombinant proteins VP1u(WT, P133R, D154A, H153A, Y157F, Y168F, D175A, D195A, Y157F/Y168F, Y157F/D175A, K162R, and A207Y), 5 µg; bee venom, 10 ng.

## Results

### Mutation of Critical Conserved Amino Acids Residues in the PLA2 Motif of VP1u Abolished PLA2 Activity

Sequence analysis revealed that almost all parvoviruses contain a conserved domain of about 40 amino acids in VP1u with a conserved HDXXY motif in the catalytic site and a conserved calcium binding loop, the YXGXG motif of PLA2s. Homology analysis showed that these motifs are present in the amino acid sequence of the VP1u region spanning positions from 130 to 195 [22]. Amino acids 130(Tyr), 132(Gly), 134(Gly), and 154(Asp) are thought to be important for calcium binding, whereas 153(His), 157(Tyr), 168(Tyr), 174(Ala), 175(Asp), and 195(Asp) were proposed to form the catalytic network for enzymatic activity and 162(Lys) is believed to be associated with phospholipid binding [13].

**Figure 1:**
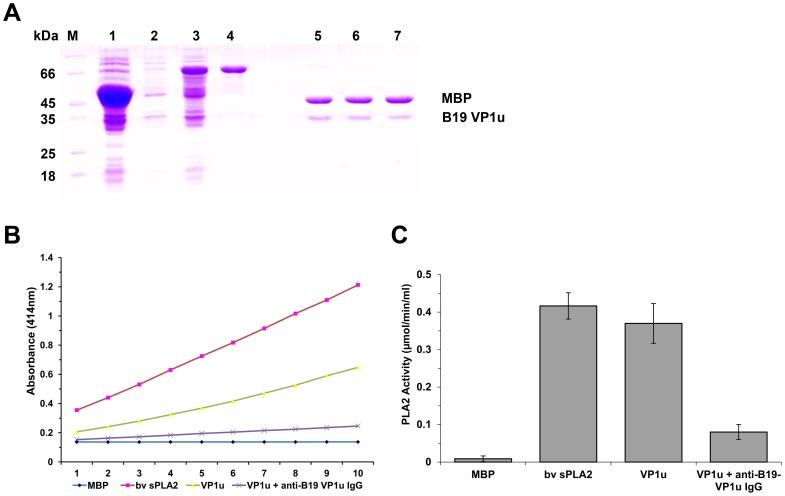
Purified VP1u proteins and PLA2 activity. **(A)** **SDS-PAGE analysis of expressed fusion protein and the purified protein VP1u or mutants cleaved by Factor Xa digestion.** M: Protein marker; lane 1: Total protein of E. coli DH5α transformed with pMAL-c_2_x with IPTG induction; lanes 2 and 3: Total protein of E. coli DH5α transformed with pMAL-VP1u with or without IPTG induction, respectively; lane 4: Purified fusion protein MBP-VP1u; lanes 5, 6, and 7: Purified VP1u, VP1u-H153A, and VP1u-D195A digested by Factor Xa. **(B–C)** **PLA2 activity on various substrates as indicated at the bottom.** The PLA2 activity is shown as a value of the absorbance at 414 nm at various time points. Purified MBP, 5 µg; bee venom, 10 ng; purified VP1u (5 µg); VP1u (5 µg) with anti-B19 VP1u (5 µg). Data are representative of three independent experiments.

To determine whether the VP1u protein expressed in *E. coli* exhibited the PLA2 activity, we first purified VP1u protein (Figure 1A). Both the purified VP1u proteins and bee venom PLA2 (positive control) showed PLA2-like activity. When the purified VP1u protein was treated with anti-B19-VP1u antibody, the PLA2 activity was decreased significantly (Figure 1B and C).

To further determine the role of the critical amino acid residues within the PLA2 motif of VP1u, a series of mutants (Group I: VP1u(Y130A), VP1u(G132A), and VP1u(D154A); Group II: VP1u(H153A), VP1u(Y157F), VP1u(Y168F), VP1u(D175A), VP1u(D195A), VP1u(Y157F/Y168F), and VP1u(Y157F/D175A); Group III: VP1u(K162R); Group IV: VP1u(P133R), and VP1u(A207Y)) were generated, and their PLA2 activities were measured. The results are shown in Table 1. All mutants in the groups showed a very low level or no PLA2 activity except that at the 195 site, in which Asp was mutated into Ala. At this site, the PLA2 activity was increased more than 10% compared with wild-type (WT) VP1u. These results were consistent with those in a previous report [23]. We also mutated two amino acids VP1u(P133R) and VP1u(A207Y) outside of the conserved region to examine whether these mutated amino acids affect the PLA2 activity. The results showed that the P133R mutant still had the PLA2 activity as expected. However, the A207Y mutant completely lost its PLA2 activity. Since this amino acid is located at the C terminal of the VP1u domain, it is possible that there is an important motif in this region.

### Amino Acids Outside the PLA2 Motif of VP1u are also Important for PLA2 Activity

**Figure 2:**
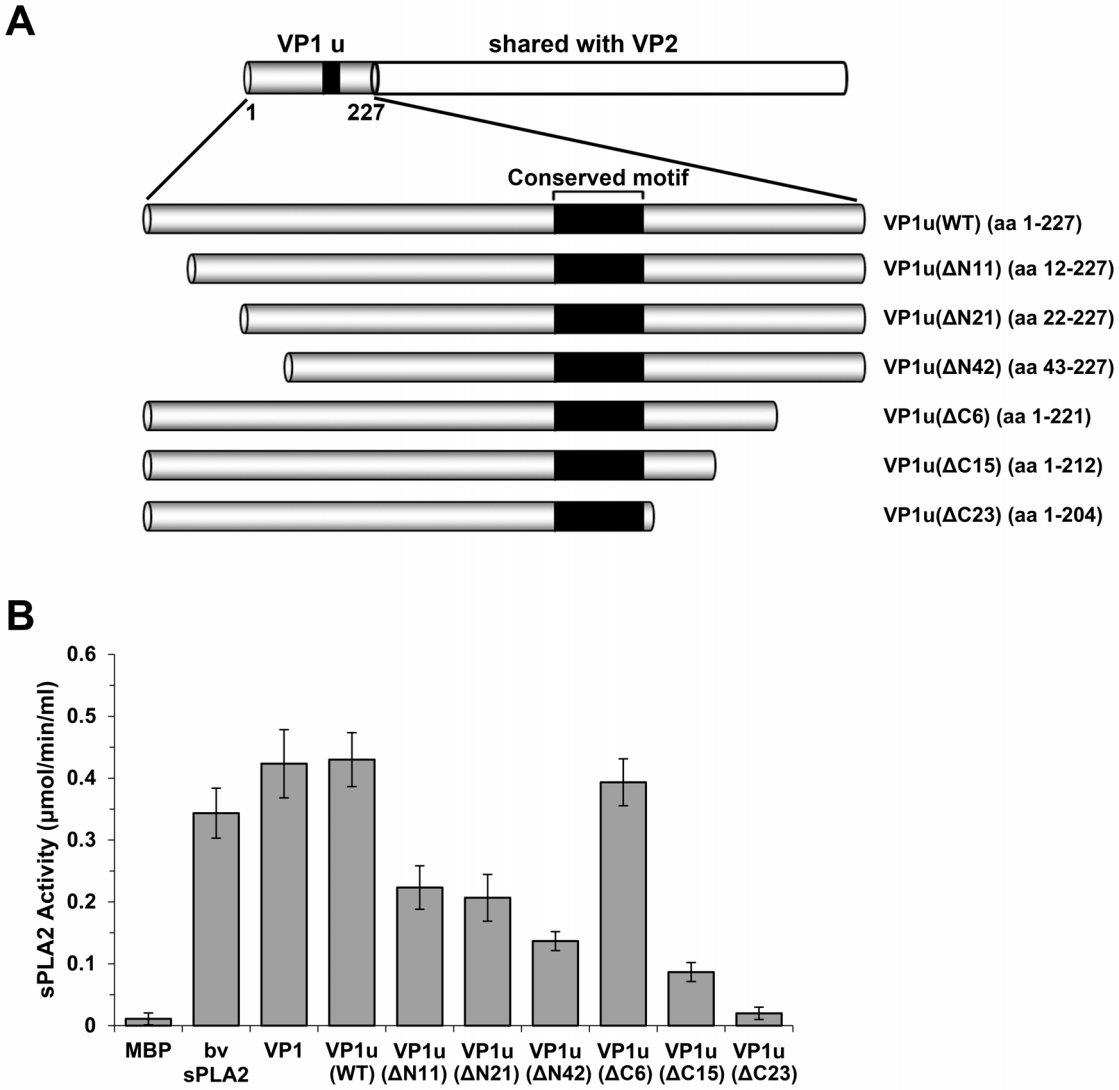
PLA2 activity of different truncated VP1 mutants. **(A) Schematic representation of the truncated VP1u region.** Six plasmids were constructed to express the C-terminal and N-terminal truncated proteins. The amino acid numbers of the proteins are shown in parentheses. The VP1u region is illustrated at the top. **(B) The PLA2 activity of different truncated VP1u proteins.** Truncated VP1u proteins, 10 µg of each; bee venom, 10 ng. Data are representative of three independent experiments.

**Figure 3:**
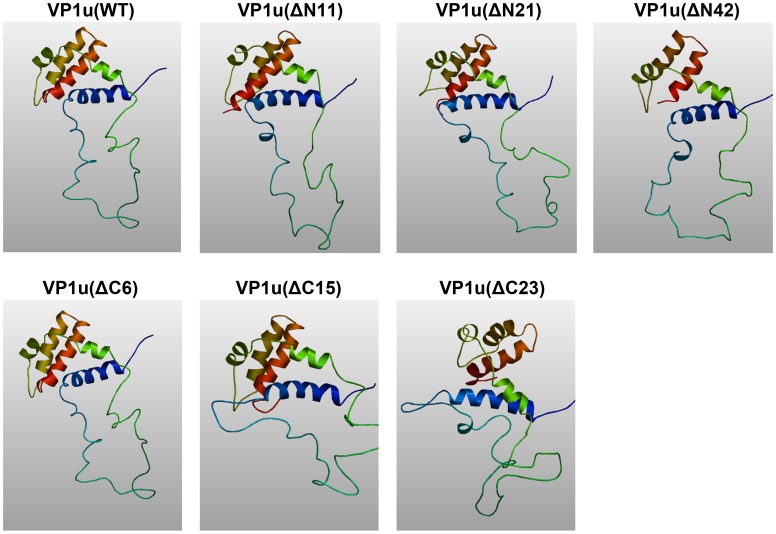
Predicted 3D structure of wild type and truncated VP1u. WT and truncated VP1u amino acid sequences were analyzed by homology modulation using the (PS)^2^ program. The 3D images were generated with the program AstexViewer.

Since the parvovirus VP1u has been shown to exhibit PLA2 activity, studies were mainly focused on the influences of the VP1u PLA2 motif on the enzyme activity. The amino acid residues spanned from 130 to 195 outside the PLA2 motif [14]–[16], [19], [24]. To examine whether amino acids in non-conserved region affect PLA2 activity, we constructed three truncated VP1u plasmids (Figure 2A, VP1u(ΔC6), VP1u(ΔC15) and VP1u(ΔC23)). Compared with WT VP1u, VP1u(ΔC6) exhibited the same PLA2 activity, while VP1u(ΔC15) decreased 80% of the PLA2 activity, and no PLA2 activity was observed from VP1u(ΔC23), which has 23 amino acids truncated in VP1u (Figure 2B). Therefore, almost all of these residues were important for the PLA2 activity of VP1u except the last several amino acids. We also constructed a series of N-terminus truncated plasmids, VP1u(ΔN11), VP1u(ΔN21), and VP1u(ΔN42), as shown in Figure 2A. Compared with WT VP1u, the PLA2 activity of both the VP1u(ΔN11) and VP1u(ΔN21) mutants were decreased nearly 50% (Figure 2B), whereas the N-terminus was further truncated (VP1u(ΔN42)), only 30% of PLA2 activity remained. These results suggested that the amino acid residues outside the PLA2 motif are also important for maintaining a proper three dimensional structure that is essential for the PLA2 activity (Figure 3).

### B19V PLA2 Destroys the Integrity of the Membrane of UT7-Epo Cells

**Figure 4:**
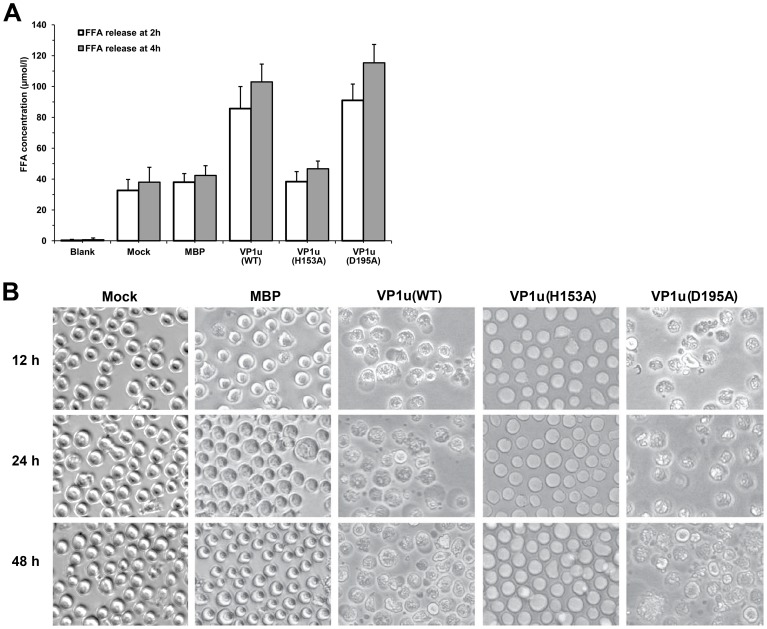
Effect of viral PLA2 on the morphology of UT/7-Epo cells. (A) Release of free fatty acid from UT7-Epo cells. Cells were treated for 2 h or 4 h with the different mutated VP1u proteins. Blank: RPMI-1640 medium; Mock: UT7-Epo cells without protein treatment; MBP, VP1u, VP1u(H153A), or VP1u(D195A): UT7-Epo cells were treated with purified MBP, VP1u, VP1u(H153A) or VP1u(D195A) proteins. Data are representative of three independent experiments. **(B) Cell morphological observation after treatment with various proteins.** Mock: normal UT7-Epo cells without protein treatment; MBP, VP1u(WT), VP1u(H153A), and VP1u(D195A): cells were treated with MBP, VP1u(WT), VP1u(H153A) and VP1u(D195A) proteins, respectively, for 12 h, 24 h or 48 h. Images were taken at a magnification of ×20 (objective lens).

Although parvovirus PLA2 was classified in the group XIII PLA2, the precise function of the viral PLA2 in the B19V VP1u is still obscure. To investigate the effects of viral PLA2 on the integrity of the cell membrane, we studied the ability of the B19V PLA2 to trigger FFA release from membrane phospholipids of UT7-Epo cells. The cells were incubated with either purified WT or mutant VP1u proteins at a final concentration of 20 µg/ml. Then, we measured the FFA concentration of the supernatant released from the treated cells after 2 h and 4 h of incubation. The results indicated that the UT7-Epo cells treated with VP1u protein released a large amount of FFA, approximately 80∼100 µmol/L (Figure 4A). There was no significant difference between the mutant VP1u(H153A) and the control. However, when cells were treated with the mutant VP1u(D195A), the FFA concentration in the supernatant was increased to approximately 120 µmol/L, which is slightly higher than the WT VP1u group, suggesting that the liberation of free acids is consistent with the PLA2 activity.

**Table:** **Table 2.** Primers used to construct B19 VP1u point mutations and truncated VP1u.

Name		Sequence (5′–3′)
**Point mutations**		
**Group I (Catalytic network)**		
VP1u(Y130A)	Forward	CCCGGTACTAACGCTGTTGGGCCTGGC
	Reverse	GCCAGGCCCAACAGCGTTAGTACCGGG
	Forward	ACTAACTATGTTGCGCCTGGCAATGAG
	Reverse	CTCATTGCCAGGCGCAACATAGTTAGT
	Forward	GCAAGGATTCATGACTTTAGGTATAGC
	Reverse	GCTATACCTAAAGTCATGAATCCTTGC
**Group II (Binding calcium ions)**		
VP1u(H153A)	Forward	GCTGCAAGGATTGCTGACTTTAGGTAT
	Reverse	ATACCTAAAGTCAGCAATCCTTGCAGC
VP1u(Y157F)	Forward	ATGACTTTAGGTTTAGCCAACTGGCT
	Reverse	AGCCAGTTGGCTAAACCTAAAGTCATG
VP1u(Y168F)	Forward	GGAATAAATCCATTTACTCATTGGACT
	Reverse	AGTCCAATGAGTAAATGGATTTATTCC
VP1u(D175A)	Forward	TGGACTGTAGCAGCTGAAGAGCTTTTA
	Reverse	TAAAAGCTCTTCAGCTGCTACAGTCCA
VP1u(D195A)	Forward	CAAGTAGTAAAAGCCTACTTTACTTTA
	Reverse	TAAAGTAAAGTAGGCTTTTACTACTTG
**Group III** **(Phospholipid environment)**		
VP1u(K162R)	Forward	AGCCAACTGGCTAGGTTGGGAATAAATCCA
	Reverse	TGGATTTATTCCCAACCTAGCCAGTTGGCT
**Group IV (No-conserved)**		
VP1u(P133R)	Forward	AACTATGTTGGGCGTGGCAATGAGCTA
	Reverse	TAGCTCATTGCCACGCCCAACATAGTT
VP1u(A207Y)	Forward	GCTGCCCCTGTGTACCATTTTCAAGGA
	Reverse	TCCTTGAAAATGGTACACAGGGGCAGC
**Wild type and truncated VP1u**		
VP1u(WT)	Forward	CGGGATCCATGAGTAAAGAAAGTGGCAAATG
	Reverse	CCCAAGCTTCCTGCAGAATTAACTGAAGTCATGCT
VP1u(ΔN11)	Forward	CGGGATCCGATGATGAATTTGCTAAAGCTGT
	Reverse	CCCAAGCTTCCTGCAGAATTAACTGAAGTCATGCT
VP1u(ΔN21)	Forward	CGGGATCCCAATTTGTGGAATTTTATGAAAA
	Reverse	CCCAAGCTTCCTGCAGAATTAACTGAAGTCATGCT
VP1u(ΔN42)	Forward	CGGGATCCGATCATTATAATATTTCTTTAGA
	Reverse	CCCAAGCTTCCTGCAGAATTAACTGAAGTCATGCT
VP1u(ΔC6)	Forward	CGGGATCCATGAGTAAAGAAAGTGGCAAATG
	Reverse	CCCAAGCTTCGGCGTTGTAAGCGGGAACTTCCG
VP1u(ΔC15)	Forward	CGGGATCCATGAGTAAAGAAAGTGGCAAATG
	Reverse	CCCAAGCTTCACTTCCTTGAAAATGGGCCACAG
VP1u(ΔC23)	Forward	CGGGATCCATGAGTAA AGAAAGTGGCAAATG
	Reverse	CCCAAGCTTCGGCAGCTGCACCTTTTAAAGTAT

a All sequences are based on the sequence of the J35 isolate (GenBank accession no. AY386330).

b GGATCC is a restriction site of *Bam*HI, AAGCTT is a restriction site of *Hind*III.

To study the effects of the viral PLA2 on host cells, we extended the incubation time of VP1u proteins with UT7-Epo cells to 12 h, 24 h, and 48 h corresponding to the previous experiments. As shown in Figure 4B, VP1u proteins exerted an important influence on the morphology of the UT7-Epo cells. With increasing VP1u protein treatment, the cells became large and round in morphology compared with control cells, and subsequent cell disruption or death occurred. No difference was observed between the VP1u(H153A) mutant and the control groups. However, UT7-Epo cells treated with VP1u(D195A) mutant showed obvious morphological changes, including cell death and cell lysis. These results suggested that active viral PLA2 is able to destroy the membrane integrity of UT7-Epo cells. These findings were consistent with the PLA2 activity and FFA results for the VP1u(D195A) mutant.

### Cellular Localization Observation of the WT and Mutant VP1u Proteins

**Figure 5:**
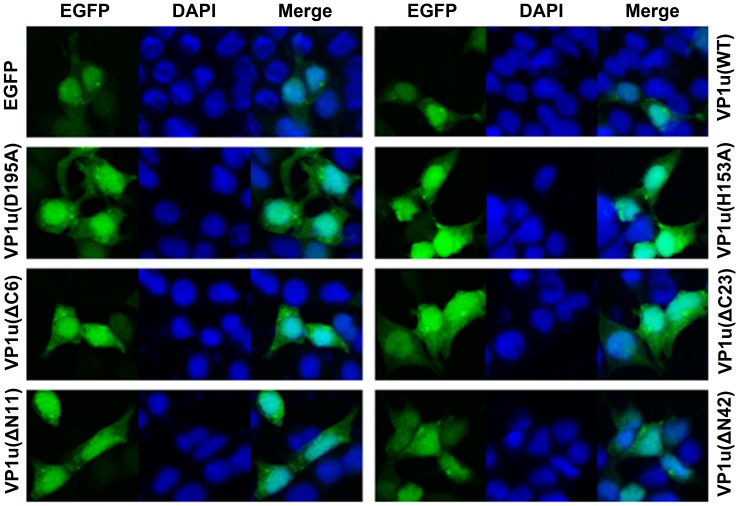
Cellular localization of wild type and mutant VP1u. HEK293T cells grown on cover glass were transfected with indicated plasmids; cells were fixed and analyzed by confocal microscopy at 48 h post-transfection. Green fluorescence showed the distribution of EGFP or EGFP-fused VP1u proteins. Nuclei were stained using DAPI. Confocal images were taken at a magnification of 40×.

B19V VP1 and VP2 proteins were previously found to be mainly localized in the nuclei [25]. However, when a point mutation was introduced at the position of amino acid 153 (H153A), the mutated VP1 protein revealed both nuclear and cytoplasmic distribution [26]. There is no report thus far on the localization of the VP1u protein when it is individually expressed in mammalian cells. We proposed that this point mutation affects putative nuclear localization signals (NLS). To confirm our hypothesis, we subsequently examined the cellular distribution of the proteins with a point mutation in conserved amino acids and the truncated mutant of VP1u. The point and truncated mutants of VP1u were expressed in pEGFP-C1 vector as EGFP-fused proteins (i.e., EGFP-VP1u(WT), VP1u(H153A), VP1u(D195A), VP1u(ΔN11), VP1u(ΔN42), VP1u(ΔC6), and VP1u(ΔC23)) in HEK293T cells. Compared to the EGFP-expressing control cells, all the EGFP-fused point-mutated and truncated proteins were localized both in the nucleus and cytoplasm (Figure 5). These results indicated that the point and truncated mutations in VP1u did not affect the distribution of the VP1 protein.

### Transcriptional Activation Effects of the Mutant VP1u Proteins on NF-κB, GAS, CRE and TNF-α Signal Pathways

**Figure 6:**
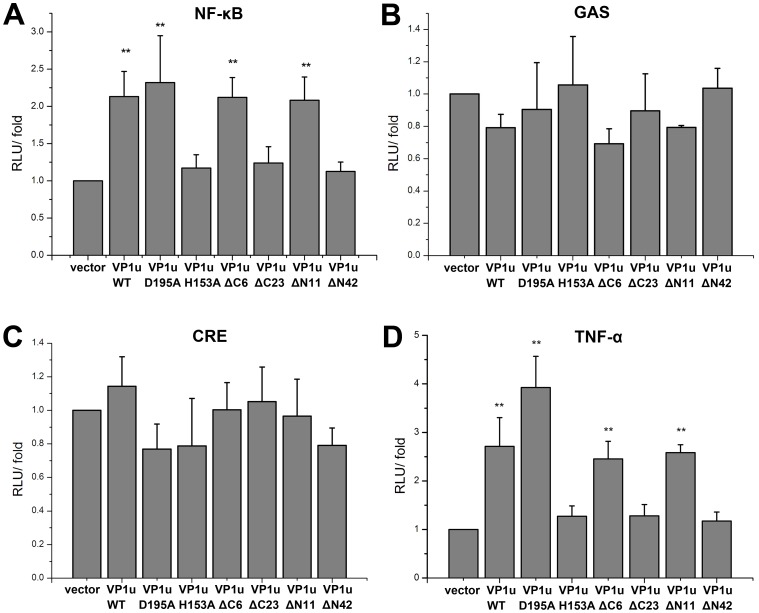
Transcriptional activation effects of mutant VP1u on NF-κB, GAS, CRE and TNF-α signal pathways. HEK293T cells were co-transfected with reporter plasmid DNA and the indicated WT- or mutant VP1u-expressing plasmids. pcDNA3.1 vector was used to adjust the transfected DNA in a total amount of 200 ng. Luciferase activity was determined at 48 h post-transfection. Data are representative of three independent experiments. An asterisk marker indicates a significant difference in the luciferase activity between the control and tested groups.

To investigate whether the VP1u protein expression affect NF- κB, GAS and CRE pathways activations, pNF-κB-luc, pCRE-luc and pGAS-TA-luc plasmids which contained luciferase reporter gene were transiently co-transfected with mutant VP1u-expressing constructs into HEK293T cells. As shown in Figure 6A, compared to the negative control vector, the VP1u(WT) and VP1u mutants FVP1u(D195A), VP1u(ΔC6), and VP1u(ΔN11) induced the NF-κB transcriptional activity increased by 2–2.5 fold. However, VP1u(H153A), VP1u(ΔC23), and VP1u(ΔN42) proteins, which possessed no PLA2 activity, did not up-regulate the transcriptional activity of NF-κB. No significant transactivation of these VP1u mutants on the GAS and CRE response pathways was observed (Figure 6B and C).

It is known that NF-κB plays a pivotal role in transcriptional regulation of various cytokines, such as IL6, IL1β, and TNF-α that are involved in triggering inammatory responses [27]. To investigate whether various mutated or truncated VP1u proteins stimulate TNF-α expression, HEK293T cells were transfected with Flag-VP1u WT, various mutant VP1u-expressing constructs, together with the reporter pTNF-α-luc, followed by an assessment of the luciferase activity. The results showed that the VP1u proteins expressed from VP1u(WT), VP1u(D195A), VP1u(ΔC6), and VP1u(ΔN11) constructs up-regulated the activity of TNF-α promoter; however, no significant increase in the TNF-α promoter activity was found in groups transfected with VP1u(H153A), VP1u(ΔC23), or VP1u(ΔN42) constructs (Figure 6D).

## Discussion

B19 is not only the etiologic agent of erythema infectiosum in children but also causes several clinical manifestations in immunocompromised patients and the patients with a high turnover rate of red blood cells, and more importantly, hydrops fetalis in pregnant woman [2], [4], [7]. Cellular signaling pathways triggered by binding of erythropoietin (Epo) to its receptor (EpoR) are critical to B19V infection of *ex vivo*-expanded erythroid progenitor cells [28]. B19V infection also induces apoptosis and cellular DNA damage response [29]–[31].

A conserved motif (HDXXY) with PLA2 activity was identified in the N-terminal unique region of the VP1 capsid protein in 30 different parvoviruses [14], [32], including B19. Parvovirus PLA2, classified as a group XIII PLA2, was shown to be responsible to mediate efficient transfer of the viral genome from late lysosomes to the nucleus for initiating viral replication, but the precise function of viral PLA2 of B19 VP1u remains elusive. Although PLA2 has an important function for B19V, the previous research was mainly focused on the conserved domain extended from amino acids 130 to 195. Mutation in the PLA2 motif located at the B19 VP1u caused a complete loss in enzymatic activity and viral infectivity [33]. In this study, we demonstrated that mutation of conserved amino acid dramatically reduced the PLA2 activity, confirming the importance of the conserved domain for the PLA2 activity. Notably, mutations of non-conserved amino acids also reduced the PLA2 activity. We found that amino acid residues outside the conserved PLA2 motif are also critical for the PLA2 activity, suggesting that the point mutation in this non-conserved region affects the protein conformation. The 3D images that were generated with the (PS)^2^ program showed the predicted structures of the WT and mutated VP1u proteins (Figure 3). There were no obvious differences between WT VP1u and truncated VP1u(ΔN11), VP1u(ΔN21) and VP1u(ΔC6) structures; however, the truncated VP1u(ΔN42), VP1u(ΔC15) and VP1u(ΔC23) had significant structural differences compared with WT VP1u, indicating that these mutations may affect the stability of VP1u. The intact VP1 showed similar PLA2 activity compared with VP1u only (Figure 2B). A previous study also indicated that the VP1u lied on the outer surface of both empty capsid and infectious virions [34]. Therefore, it is possible that PLA2 activity of VP1u is independent of the B19 capsid structure, as the flexible VP1u structure in the capsid of human parvovirus adeno-associated virus (AAV) [35], but plays a key role in viral infection, which is expected to be an efficient target for neutralizing antibody.

It has been reported that the VP1u of porcine parvovirus induced the release of FFA from CHO-K1 cells, but not the B19V VP1u [33]. Our results showed that the WT B19V VP1u induced FFA release in UT7-Epo cells, while the VP1u(H153A) mutant protein did not. Notably, the VP1u(D195A) mutant protein even increased the FFA concentration in supernatant more than the WT VP1u did. The results were consistent with those of the morphology alteration of the UT7-Epo cells treated with the VP1u proteins. With increasing treatment by WT VP1u or VP1u(D195A) proteins, the cells appeared large and round, subsequently followed by cell disruption or death; however, there was not significant change in the VP1u(H153A) group, suggesting that the cell morphology change correlates well with the PLA2 activity induced by the VP1u proteins. This result indicated that the PLA2 activity plays a direct role in initiating the inflammatory response during B19 infection of UT7-Epo cells. To prevent the contamination of the UT7-Epo cells by LPS, the MBP protein purified from bacteria was incubated with the cells as a negative control. The result showed there were no FFA detection in supernatant and no cell morphology change (Figure 4).

The B19 capsid protein VP1 and VP2 were mainly localized in the nuclei [25]. The VP1(H153A) protein revealed a nucleocytoplasmic distribution. The point mutation of H153A probably affects the presentation of the putative NLS. To investigate the effects of the point mutation and truncated VP1u on its localization in cells, we utilized the EGFP-fused VP1u to determine VP1u distribution in cells. Our results indicated that both the WT and the mutant VP1u proteins revealed a nucleocytoplasmic distribution, suggesting that these mutations did not change the localization of VP1u.

B19 infection has been suggested as a cause of autoimmune diseases that involve inflammatory cytokines. TNF-α, a major inflammatory cytokine, plays an important role in the pathogenesis of systemic autoimmune diseases [36], [37]. In a consistency, our results showed that B19 VP1u and its PLA2 activity are associated with the induction of TNF-α, which is dependent on the stimulation of the NF-κB pathway. To investigate the signaling pathway involved in the production of cytokines, GAS transcriptional activation was examined. The results showed that there was no difference between the WT and various VP1u mutants in GAS luciferase activity. A similar result was also observed in CRE luciferase activity, suggesting that the WT and VP1u mutants that contain the PLA2 activity induce the production of TNF-α through the NF-κB signaling pathway, but cannot activate the GAS- and CRE- pathways.

The PLA2 is known to play important roles in many inflammatory processes and the immune response; however, the roles of B19V VP1u and its PLA2 motif on B19-caused diseases have not been investigated. Our preliminary results suggest that amino acid residues other than the PLA2-conserved motif are critical for the PLA2 activity. Understanding the crucial function of B19V PLA2 in the invasion of B19 in the host cells will provide clues for further elucidating the role of B19 VP1u in the host response to B19 infection and B19-caused diseases.

## Materials and Methods

### Cell Culture

UT7-Epo cells, which have been previously reported to have an increased sensitivity for parvovirus B19, were kindly provided by Dr. Peter Tijssen. The cells were maintained in DMEM (Wisent) containing 10% fetal calf serum (FCS), 2U/ml recombinant human erythropoietin (Epo) erythropoietin and antibiotics, and were grown at 37°C with 5% CO_2_. HEK293T cells were maintained in DMEM (Wisent) containing 10% fetal calf serum in an atmosphere of 5% CO_2_ at 37°C. Transient transfections were performed using jetPEI according to the manufacturer’s instructions (Polyplus).

### Plasmids and Site-directed Mutagenesis

A 681-bp DNA fragment of the B19 genome (AY386330) was amplified by PCR using primers 5′-CGGGATCCAGTAAAGAAAGTGGCAAATGG-3′ (forward) and 5′-CCCAAGCTTTTAGCTTGGGTATTTTTCTGAGGC-3′ (reverse), in which a *Bam*HI site at the 5′ end and a *Hind*III site at the 3′ end were introduced for cloning it into pUC-18a vector. Based on the pUC-VP1u plasmid, the point mutations were introduced into the VP1u (Table 2), respectively, using a site-directed mutagenesis strategy. To construct Flag-mVP1u and GFP-mVP1u, point mutations of the conserved amino acids were amplified and cloned into the *Eco*RI and *Hin*dIII sites of the pCMV-Flag vector and the *Eco*RI and *Bam*HI sites of pEGFP-C1 vector (Clontech), respectively. All mutations were confirmed by sequencing.

### Preparation of Recombinant Human B19 VP1u Protein and Mutation Proteins

The full-length VP1u region was digested from plasmids (pUC-VP1u, pUC-VP1u-H153A, and pUC-VP1u-D195A) and inserted into the prokaryotic expression vector pMAL-c2X to generate plasmids pMal-VP1u, pMal-VP1u-H153A and pMal-VP1u-D195A. To construct the truncated VP1u mutants, different PCR fragments were inserted into the pMAL-c2X (NEB) vector by using *Bam*HI and *Hind*III sites (Table 2). All the clones were confirmed by sequencing and then expressed in the *E. coli* DH5α strain. The fusion protein was induced by IPTG in the *E. coli* DH5a cells, analyzed by SDS-PAGE and Western-blot. Then, the target fusion protein was purified using amylose affinity chromatography. The MBP tag was later cleaved from the fusion protein by Factor Xa.

### PLA2 Catalytic Activity Assay

The PLA2 activity of VP1u proteins and mutants (P133A, D154A, H153A, Y157A, D175A, D195A, Y157F\Y168F, and Y157F\D175A, K162R and A207Y) were assayed using the PLA2 Activity Kit (Cayman Chemical, Ann Arbor, MI, USA) according to the manufacturer’s instructions. Upon hydrolysis of the thioester bond at the *sn*-2 position by PLA2, free thiols were detected using 5,5′-dithio-bis-(2-nitrobenzoic acid) (DTNB) with dynamic colorimetric measurements at a wavelength of 414 nm every minute for 10 min. The VP1u and its mutants (5 µg each) and truncated mutants (10 µg each), as well as a positive control of bee venom PLA2 (10 ng), were tested three times and analyzed at five different time-points. Results are expressed as micromoles per minute per milliliter.

### Release of Free Fatty Acids (FFA) from UT7-Epo Cells and Changes of Cell Membrane

UT/7-Epo cells were cultured in 24-well plates in 0.5 ml of medium per well. When cells reached ∼60% confluence, VP1u proteins were filtered with 0.45 µm filter, then added into cell culture at a final concentration of 20 µg/ml. After incubation at 4°C for 2 h or 4 h, 50 µl supernatant was collected by centrifugation of the cells at 3,000 rpm for 10 minutes. Then the supernatant was assayed for FFA concentration by using a colorimetric assay (Ultrasensitivity assay kit for free fatty acids, Applygene Technologies Inc.), as described by the manufacturer’s instructions, with dynamic colorimetric measurements (the optical density at 550 nm). For cell morphology observation, the UT7-Epo cells were incubated with various VP1u proteins for 12 h, 24 h, 48 h at a final concentration of 20 µg/ml; the cells were observed under an optical microscope.

### Cell Transfection and Luciferase Assay

HEK 293T cells were maintained in Dulbecco’s modified Eagle’s medium (Hyclone) supplemented with 10% fetal bovine serum. Cells were transfected using jetPEI™ (Polyplus-transfection) according to the manufacturer’s protocol. To measure respectively the NF-κB, CRE and the TNF-α promoter activity, 293T cells were seeded into 96-well plates and then transiently co-transfected with the indicated reporter plasmids pNF-κB-luc, pCRE-luc, pGAS-TA-luc or pTNFα-p-luc containing the promoter region of human TNF-α. After 24 h, cells were harvested, and the luciferase activity was measured using the Luciferase reporter assay system (Promega). The reporter NF-κB-luc (Clontech) contains four copies of the κB response element, the reporter pGAS-TA-luc (Clontech) contains two copies of the STAT1 enhancer element, and the reporter pCRE-Luc (Stratagene) contains four copies of a consensus CRE. The primers used for human TNF-α promoter were as follows: sense primer, 5′-CGGGGTACCAGCTCCTGGGAGATATGGC-3′, and anti-sense primer, 5′-CCCAAGCTTGGGTGTGCCAACAACTGC-3′. The TNF-α promoter PCR products were cloned into the pGL3-basic vector through *Kpn*I/*Hind*III sites as pTNF-α-P-luc.

### Confocal Microscopy

HEK293T cells grown on cover glass were transfected with indicated plasmids expressing GFP-tagged VP1u/mVP1u/ΔVP1u. After 48 h of transfection, cells were washed in PBS and fixed in 4% paraformaldehyde for 20 min at 37°C. The washed cells were incubated with DAPI solution (0.5 µg/ml) for 20 min at room temperature. After three washes in PBS, cells were analyzed using a Zeiss LSM700 laser confocal microscopy system.
